# Robotic surgery of the urothelial carcinoma of the upper urinary tract single surgeon initial experience, 66 consecutive cases

**DOI:** 10.1186/s12894-024-01629-y

**Published:** 2024-11-01

**Authors:** Mahmoud Farzat, Sami-Ramzi Leyh-Bannurah, Florian M. Wagenlehner

**Affiliations:** 1 Department of Urology and Robotic Urology, Diakonie Klinikum Siegen, Siegen, Germany; 2 Department of Urology, Pediatric Urology and Andrology, Justus-Liebig University of Giessen, Giessen, Germany; 3 Martini Clinic, Prostate Cancer Center at University Hospital Hamburg-Eppendorf, Hamburg, Germany

**Keywords:** Upper urinary tract carcinoma (UTUC), RANU, RARC, Segmental ureterectomy

## Abstract

**Purpose:**

Robotic surgery is increasingly utilized in the treatment of urothelial carcinoma of the upper urinary tract (UTUC). This study investigates the advantages and burden of robot-assisted surgical treatment of the urothelial carcinoma of the upper urinary tract in a referral urological department, along with their functional and oncological results.

**Methods:**

The study included 66 prospectively enrolled patients who were surgically treated by a single, robotically specialized surgeon between July 2019 and December 2023. Patients were divided into three groups. Group 1: 50 patients underwent robot-assisted radical Nephroureterectomy (RANU) with bladder cuff excision, Group 2: 11 patients underwent RANU simultaneously with robot-assisted radical cystectomy (RARC), and Group 3: 5 patients underwent robot-assisted segmental ureterectomy (RASU). Clinical and oncological parameters were compared. Perioperative morbidity according to Clavien-Dindo was the primary endpoint of our study. The secondary endpoint was oncologic outcomes.

**Results:**

37.8% of patients had locally advanced carcinomas. The average console time of RANU with bladder cuff excision was 69 min. The rate of positive surgical margins was *n* = 1/66 (2%). Lymphadenectomy (LAD) was performed on 30% of patients, with a mean of 13.7 lymph nodes removed. Of those who received LAD, 33% had lymph node metastasis. *n* = 6/66 (9%) patients received blood transfusion. The overall complication rate was 24%. The readmission rate was 7.5%. With a median follow-up of 26 months, the 2-year recurrence-free survival rate was 84.4%, and the 2-year overall survival rate was 94%.

**Conclusion:**

Robotic surgery is a feasible option for treating UTUC that can be adapted to meet the surgical needs of each patient. Prospective studies are warranted to confirm its benefits.

## Introduction

Upper urinary tract carcinoma (UTUC) accounts for approximately 7% of all urothelial cancers [1]. Previous series mostly focused on either comparison between open versus laparoscopic radical nephroureterectomy (ONU vs. LNU) [2–4] or laparoscopic versus robotic nephroureterectomy (RANU) [5–8]. Simon et al. found in their randomized trial that LNU patients profited from the minimally invasive approach regarding hospital stay and blood loss while the oncological control in advanced-stage diseases was in favor of ONU [9]. Since LNU is a challenging procedure concerning bladder cuff excision, many surgeons tended to combine the laparoscopic approach with an open ureterectomy [10]. However, Peyronnet et al. suggested that oncological outcomes of LNU may be less favorable than those of open radical nephroureterectomy (RNU) when the bladder cuff is excised laparoscopically, particularly in patients with locally advanced high-risk tumors [4]. Also, the impact of lymphadenectomy (LAD) on clinical outcomes during radical nephroureterectomy (RNU) was also investigated by various authors [11–13]. For example, Yoo et al. found that LAD may impact the 5-year recurrence-free survival [14]. In consequence, a lymphadenectomy should be ideally performed during RNU. Second, in patients with low-risk / low-grade and noninvasive UTUC a kidney-sparing approach represents an important therapeutic option [15]. In this context, the robot-assisted approach might mitigate potential disadvantages of the laparoscopic approach and provide a solution for adequate oncological outcomes, sufficient lymphadenectomy and kidney sparing in select patients. Data is still very limited, but RANU is increasingly utilized in the surgery of UTUC [16, 17]. For example, Yajima et al. reported a case of simultaneous RANU with robot-assisted radical cystectomy (RARC) [18]. Thus, we investigated the complications and oncological outcomes of robot-assisted radical nephroureterectomy (RANU) and robot-assisted segmental ureterectomy (RASU) patients in a referral urological department performed by a single robotic expert surgeon.

## Methods

We performed 66 consecutive procedures transperitoneally with the Da Vinci X^®^ Surgical System (Intuitive Surgical, Sunnyvale, CA, USA). Preoperatively, all patients underwent a ureteroscopy, and biopsies were performed when possible, except in very few symptomatic cases where the tumors were clearly visible on CT scans. However, only 20% of cases showed positive biopsies with evidence of carcinoma. Patients with suspicion of lymph node metastasis or locally advanced tumors in the preoperative staging CT scan or MRI who were willing and whose kidney function allowed it received neoadjuvant chemotherapy. Surgeries were performed by a single robotic expert surgeon with a caseload of over 2000 combined robotic procedures. All RANU patients were positioned in a lateral position. A Capnoperitoneum of 8 mm/Hg was established through a pararectal mini-laparotomy, which was used to extract the specimen. Four robotic arms were utilized. After finishing the kidney part, the robotic instruments were reassigned for the ureteral part without the need for Re-docking or patient repositioning. In two patients, one with ureter-duplex and the other with a locally advanced distal ureteral tumor with iliac vessel involvement., a repositioning was needed to continue the procedure. All RASU and RARC patients were operated in modified lithotomy position. 20 Fr urethral Foley catheter was inserted. In RARC patients ports are placed transperitoneal similar to a robotic radical prostatectomy port configuration, only more cephalad. Hilar, paraaortic, retrocaval and interaortocaval lymphadenectomy was performed in kidney pelvis tumor cases, in which lymph nodes were deemed suspicious on preoperative CT scans or when tumours met high-risk criteria according to EAU guidelines for UTUC [1]. In case of high-risk ureteral urothelial cancers, the iliac lymph nodes were removed. Group 1 was defined as RANU patients. They also received a bladder cuff excision, and no drain was inserted. Group 2 was defined as RANU patients with simultaneous RARC. In those, the resected kidney was removed in an en-bloc approach with the bladder and a drain was inserted in this group. Group 3 was defined as RASU patients, i.e. distal ureterectomy, who received a catheter insertion and a bladder closure. These patients did not receive any drains. In group 1 and 3, a cystography was performed on the third day after surgery. When cystography was uneventful, patients received mitomycin and the catheter was removed. Overall, 66 patients underwent RANU and RASU between July 2019 and December 2023. We compared demographic and perioperative parameters between groups. Postoperative complications were graded using the Clavien-Dindo classification [19]. Follow-ups were performed regularly according to EAU guidelines [1].

Data was collected prospectively in an institutional database and analyzed with SPSS^®^ v27. Categorical variables were presented as frequencies, while continuous variables were presented as mean values. Kolmogorov-Smirnov test verified normal distribution. Independent T-test and Mann-Whitney U test were used for matched-pair analysis of parametric and non-parametric variables, respectively. Pearson’s chi-square test was used to compare relative frequencies. For parametric numeric variables, a one-way ANOVA test was performed, followed by a post hoc comparison (Bonferroni) test if needed. The independent samples Kruskal-Wallis test was used for nonparametric variables.

The study was conducted under the ethical standards of the Declaration of Helsinki and approved by the ethics committees of the medical association Westfalen-Lippe and Wilhelm’s University of Muenster (2023-500-f-S).

## Results

### Baseline parameters

Patients were grouped according to surgical procedure they received for UTUC treatment (Table 1). Group 1 had 50 patients who underwent RANU, Group 2 had 11 patients who had RANU simultaneously with RARC, and Group 3 had 5 patients who underwent RASU. Overall, patients’ mean age was 71 years with similar distribution among groups (*p* = 0.7). Their average BMI was 31 with no variation among groups (*p* = 0.9). 45% of patients were classified as ASA 3 with no statistical differences observed among groups (*p* = 0.38). Group 2 patients had confirmed preoperative histological tumors, while only 20% in group 1 did (*p* = 0.014). Hence in group 1, surgery was also based on clinical and radiographic diagnosis, even with negative or equivocal endourological biopsy results. Group 2 had the most advanced tumours, with 80% of them being at least clinical T2 tumors (*p* = 0.014). Group 1 had the highest intake of anti-coagulation medication, with 15 (33%) patients taking aspirin (*p* = 0.003). Overall, *n* = 8/66 (12%) patients received neoadjuvant platin-based chemotherapy. All other study parameters were similar between the groups. Further details are given in Table 1.

**Table 1:** Analysis of demographic and baseline characteristics

UTUC	Total (*N* = 66)	Group 1 RANU *N* = 50 (77%)	Group 2 Combined RANU and RARC *N* = 11 (16%)	Group 3 Robot-assisted segmental Ureterectomy *N* = 5 (7, 6%)	*p*-Value
Age (years), mean	71	72	69	70	0.7
BMI (kg/m^2^), mean	31	31	32	31	0.9
ASA-score					0.4
1 2 3	20 (30) 16 (24) 30 (45,5)	14 (28) 11 (22) 25 (50)	5 (45) 3 (27) 3 (27)	1 (20) 2 (40) 2 (40)	
Preoperative Hgb (g/dl), mean	12.2	12.2	11.8	12.8	0.7
Tumor location					
Intramural ureter Other parts of Ureter Kidney pelvis Multifocal	12 (18) 18 (27) 34 (51) 2 (3)	3 (6) 12 (24) 33 (66) 2 (4)	9 (81) 1 (9) 1 (9) 0	5 (100) 0 0 0	
Preoperative Histology					
No Histology Tis Ta T1 T2 T3 T4	44 (66) 1 (1,5) 11 (16) 1 (1,5) 8 (12) 0 1 (1,5)	40 (80) 0 9 (18) 1 (2) 0 0 0	0 1 (9) 1 (9) 0 8 (72) 0 1 (9)	4 (80) 0 1 (20) 0 0 0 0	*0.014*
Neoadjuvant Chemotherapy					
2 cycles cisplatin-Gemcitabine 3 cycles cisplatin-Gemcitabine 4 cycles cisplatin-Gemcitabine 6 cycles cisplatin-Gemcitabine	2 (3) 1 (1,5) 2 (3) 3 (4,5)	0 1 (2) 0 1 (2)	1 (9) 1 (9) 2 (18) 2 (18)	0 0 0 0	0.001
Anti-coagulation Aspirin NOAC	0 19 (13,6) 6 (9)	0 15 (30) 3 (6)	0 3 (27) 0	0 1 (20) 3 (60)	*0.003*
Time from first diagnosis to procedure (months), mean	1.3	1.27	1.1	2	0.5

Categorical data are presented as numbers %, UTUC: Upper Urinary Tract Urothelial Cell Carcinoma, RANU: robot-assisted nephroureterctomy, RARC: robot-assisted radical cystectomy. BMI: body mass index, ASA: American Association of Anesthesiology Morbidity Score, Hgb: hemoglobin, NOAC: new oral anticoagulants

### Intra- and perioperative data

Overall, the average console time was 63 min (Table 2). Group 2 patients, who received RANU simultaneous with RARC, had the shortest time at 42 min for the RANU procedure vs. the longest time in RASU patients at 73 min (*p* = 0.065 ). Overall, 38% of patients had locally advanced carcinomas and statistical analysis showed no significant difference for tumor stage between groups (*p* = 0.18). 62% of patients had high-risk carcinomas (*p* = 0.3), and only one patient in the study had a positive surgical margin. Among those, who received a LAD (*n* = 20), the mean number of lymph nodes removed was 13.7, with a maximum mean number of 22 in group 2, (*p* = 0.035). In the same group of those, who received a LAD, 6 (33%) were positive for LN metastases. The mean hospital stay for all patients was 8.1 days, with the longest stay in group 2 at 10 days, which was statistically significantly longer compared to other study groups (*p* = 0.4). Overall, 6 out of 66 patients (9%) received a perioperative blood transfusion, with no significant difference between the study groups (*p* = 0.6). More details in Table 2.

**Table 2:** Intra- and postoperative data and pathological findings between groups

UTUC	Total (N = 66)	Group 1 RANU N 50 (77%)	Group 2 Combined RANU and RARC N = 11 (16%)	*Group 3* Robot-assisted segmental Ureterectomy *N = 5 (7,6%)*	p-Value
Console time (minute), mean (SD)	63 (34)	69 (37)	42 (15)	73 (12)	0.065
Pathological tumor stage, n (%)*					
pT0	5 (7,5)	5 (10)	0	0	0.18
pTa	11 (16)	9 (18)	0	2 (40)	
pT1	12 (18)	11 (22)	0	1 (20)	
pT2	13 (19,6)	10 (20)	2 (18)	1 (20)	
pT3	21 (32)	15 (30)	5 (45)	1 (20)	
pT4	4 (6)	0	4 (36)	0	
Urothelial carcinoma grade*, n (%)					
0	5 (7,5)	5 (10)	0	0	0.3
1	14 (21)	11 (22)	0	3 (60)	
2	5 (7,5)	5 (10)	0	0	
3	42 (63)	29 (58)	11 (100)	2 (40)	
Postoperative Chemotherapy					
(yes. vs. none), n (%)					0.3
2 cycles cisplatin-Gemcitabine, n (%)	2 (3)	0	2 (18)	0	
3 cycles cisplatin-Gemcitabine, n (%)	1 (1,5)	1 (2)	0	0	
4 cycles cisplatin-Gemcitabine, n (%)	1 (1,5)	0	1 (9)	0	
6 cycles cisplatin-Gemcitabine, n (%)	1 (1,5)	0	1 (9)	0	
Initiation of CI therapy, n (%)	3 (4,5)	1 (2)	1 (9)	1 (20)	
Positive surgical margins (total), (%)	1 (1,5)	0	1 (9)	0	0.8
Number of patients, who received a lymphadenectomy, n (%)	20 (30)	10 (20)	9 (81)	1 (20)	0.001
Number of lymph nodes removed in patients, who received a lymphadenectomy, mean (SD)	13.7 (13.7)	7 (10)	22 (13)	2 (0)	0.035
Number of patients, who had a lymphadenectomy and had metastases among the total number of surgically treated patients	6/20 (33%)	1/10 (10%)	5/9 (55%)	0/1	0.002
Length of hospitalization (days), mean (SD)	8.1 (6.5)	7.9 (7)	10 (5.5)	5.8 (1)	0.4
Transfusion rate, n (%)	6 (9%)	4 (8)	2 (18)	0	0.6

* patients with multiple tumours: the most significant cancer was listed, Categorical data are presented as numbers %, UTUC: Upper Urinary Tract Urothelial Cell Carcinoma, RANU: robot-assisted nephroureterctomy, RARC: robot-assisted radical cystectomy. SD: standard deviation, CI: Check point inhibitor, * Grade according to WHO classification 1999 (Busch et al.) [34]

### Complications

Group 2 Patients (36%) had more complications than group 1 (24%) und group 3 (0%) (*p* = 0.04). Overall 5/66 patients (7,5%) were readmitted within 90 days after discharge. The statistical analysis showed no difference between groups (*p* = 0.8). The most common major complication we observed, was 4 incisional hernias (CDC 3b) on the mini-laparotomy site, that was used to extract the specimen. One female patient developed an embolus of the arteria iliaca externa (CDC 3b) and had to undergo an emergency embolectomy. One 90-year-old male patient experienced bleeding (CDC 3b) on the first operative day with a hemoglobin decrease of more than 6 g/dl after an uneventful intraoperative course. One male patient had a diagnostic laparoscopy due to suspicion of mechanical bowel obstruction (CDC 3b), which was not confirmed, and resolved thereafter after further medical bowel stimulation. The final complication was wound infection which necessitated wound revision and a superficial Vacuum-assisted closure system (CDC 3b). The surgery-related mortality rate at 3 months in our cohort was 3%; *n* = 2/66. Specifically, two geriatric patients over 80 years old received palliative surgery due to persistent macrohematuria, refractory to endosurgical treatments. Of those, the first, female patient died due to cardiac decompensation (CDC 5). The second, male patient had a hostile abdomen due to previous surgeries, underwent bowel adhesiolysis and bowel resection, before the robot-assisted nephroureterectomy and later died due to multiorgan insufficiency (CDC 5). More details in Table 3.

**Table 3:** Complications, readmissions and oncological long term results among study groups, follow up time-lapse 3–48 months

UTUC		Total (*n* = 66)	Group 1 RANU *N* = 50 (77%)	Group 2 Combined RANU and RARC *N* = 11 (16%)	Group 3 Robot-assisted segmental Ureterectomy *N* = 5 (7, 6%)	*p*-value
Total		16 (24)	12 (24)	4 (36)	*0*	*0.04*
Minor	**CDC I**	4 (6)	4 (8)	0	0	
	**CDC II**	1 (1,5)	0	1 (9)	0	
Major	**CDC IIIa**	1 (1,5)	1 (2)	0	0	
	**CDC III b**	8 (10,6)	4 (8)	4 (36)	0	
	**CDC VI**	0 (0)	0 (0)	0	0	
	**CDC V**	2 (3)	2 (4)	0	0	
*Readmissions*		5 (7,5)	4 (8)	1 (9)	0	0.8
Postoperative Instillation therapy Mitomycin BCG		26 (39) 1 (1,5)	25 (50) 1 (2)	0 0	1 (20) 0	
Local recurrence		1 (1,5)	1 (2)	0	0	
Bladder Recurrence		6 (9)	5 (10)	0	1 (20)	
Distant metastasis		4 (6,6)	1 (2)	3 (27)	0	
Follow up (Month), mean		25.9	24.5	28.5	33.8	0.5
Cancer related death		3 (4,5)	2 (4)	1 (9)	0	
Death to other reasons		1 (1,5)	1 (2)	0	0	
The 2 year Recurrence free survival		56 (84,4)	43 (86)	8 (72,7)	5 (100)	0.25
The 2 year Cancer specific survival		63 (95,4)	48 (96)	10 (91)	5 (100)	0.5
The 2 year Overall survival		62 (94)	48 (96)	9 (82)	5 (100)	0.6

CD: Clavien-Dindo, BCG: Bacillus Calmette–Guérin, Categorical data are presented as numbers %

### Oncological results

In group 1, in 10% of patients, a tumor could not be detected in the final pathology. Of those, 2 patients had neoadjuvant chemotherapy, 2 patients had highly suspicious tumor findings preoperatively in the multiphase contrast-enhanced CT-Scan and one young, 42-year-old, male patient had endoscopically proven ureteral cancer. Postoperatively, *n* = 26/50 (52%) patients received mitomycin. 5 patients received adjuvant platin-based chemotherapy while 3 patients received checkpoint inhibitors. The median follow-up in our series is 26 months (interquartile range from 9 to 43 months). One patient with locally advanced UTUC had local recurrence at the kidney site, while 6 (9%) patients had bladder recurrence and 4 (6%) patients had distant metastasis. We recorded 3/66 (4,5%) cancer-related deaths and 1 (1,5%) death due to other reasons. The recurrence-free survival in our study was 84,4% at 24 months. While the cancer-specific survival was 95% at 24 months, the overall survival was virtually identical at 94% at 24 months. Details are given in Table 3.

## Discussion

With the increased adoption of robotic surgery in the treatment of urothelial malignancies including UTUC, the vast majority of related literature focused on the comparison between the different surgical approaches and their results in context of UTUC [2–4]. Veccia et al. found that the robotic approach led to better tetrafecta outcomes than the laparoscopic approach [20]. Some surgeons combined different approaches, such as laparascopy and open surgery with robot-assisted surgery to gain the best possible results [6, 10]. Some investigated the potential benefit of LAD during nephroureterectomy [11]. Others developed a nomogram to predict postoperative renal insufficiency for adjuvant chemotherapy after RANU [21].

However, to this date, previous series are very sparse and mostly limited due to small sample sizes and/or still maturing surgical expertise. For example, the multicenter study by Campi et al. relied on a total combined cohort of 81, with a highly variable robotic caseload [22]. Moreover, similar to RARC patients UTUC patients represent a highly variable patient cohort, young and fit patients vs. senior patients with high comorbidity burden and previous surgical interventions [23]. Thus, we relied on a real world cohort without strict selection criteria with an adequate sample size and a single robotic-surgeon expert with a combined caseload of over 2000 combined robotic procedures (i.e. RARP, RARCs and complete or partial nephrectomies). This is reflected by three different robot-assisted surgical methods, RANU, combined RANU in en-bloc fashion and RASU. Moreover, we relied on comprehensive results, i.e. intra- and perioperative data including complications and oncological follow-up. Our study had important findings.

First, mean age of 71, a mean BMI of 31 that denotes obese patients and a majority of ASA3 status proportion overall indicate a challenging, but real-life patient cohort. Similarly, tumor characteristics are reflected in the careful choice of surgery approach.

Second, it is important to note that in our real-world cohort, 10% of patients could benefit from a kidney-sparing (i.e. RASU) approach if tumor localization and size were carefully considered. In 11 out of 66 cases (16%) with an aggressive, muscle-invasive bladder carcinoma accompanied by UTUC, a combined RANU and RARC procedure was found to be feasible.

Third, our intra- and perioperative characteristics indicate rather short surgical times and low Clavien-Dindo Complication (CDC) rates, demonstrating that the robot-assisted UTUC surgery is particularly suited with respect to perioperative morbidity for a comorbidity-burdened patient cohort. The surgical efficiency and outcomes might be attributed to two aspects. First, the surgical experience of almost 2000 robotic procedures, which is considered a super-expert [24, 25] and second, the single docking of the robot, i.e. no repositioning of the patient and no re-docking during the procedure. This notion and corresponding causality is supported by Yajima et al., who reported the first case of combined RANU and RARC in Japan. They reported RANU console time to be 66 (RANU) and 207 (RARC) minutes which is in accordance with our findings (42 min for RANU in group 2) [18]. Moreover, Kamei et al. compared the en-bloc cystectomy with radical nephroureterectomy between 17 open-surgical and 10 robot-assisted patients and found the minimally invasive approach to be non-inferior [26]. These findings clearly demonstrate the need to include surgical expertise in analyzing results of such robot-assisted surgeries as in our study at hand, going hand-in-hand with the en-bloc approach.

Fourth, in the study at hand technical feasibility included LAD, which prognostic impact remains of great debate. For example, Inokuchi et al. reported simultaneous LAD, with consistent mean LAD yield compared to our study [11]. Specifically, Dominguez-Escrig et al. reported that template-based and complete lymph node dissection improves cancer-specific survival (CSS) in patients with high-stage UTUC and reduces the risk of local recurrence [12]. On the other hand, Hakimi et al. found that LAD didn’t improve overall survival in patients with positive Lymph nodes [27]. Thus, a LAD remains an important cornerstone in UTUC surgery. The number of removed lymph nodes ranged between 5,5 and 21 [5, 14]. Yoo et al. found that 12.1% who underwent lymph node dissection, had pathological lymph node metastasis in their final pathology [14]. Similarly, De Groote et al. performed LAD in 41% of patients and found lymph node involvement in 29% [16]. In our study, the mean number of removed lymph nodes was 13.7 similar to other publications [5, 14]. Furthermore, we performed LAD only in high-risk patients and or when suspicion was given in a CT scan preoperatively. Similarly, we found metastasis in 33% of patients, who underwent LAD. Taken together, our findings are consistent with previous series and demonstrate that LAD during RANU with or without RARC is highly feasible.

Fifth, despite high comorbidity burden our cohort experienced an overall major complication rate (i.e. CDC III a or higher) of 17%. These results are lower than reported by other surgeons [5, 6, 16]. However, we did not yet apply the most recent Comprehensive Complication Index (CCI^®^) introduced by Slankamenac, adopted for open radical cystectomy by Vetterlein et al. and first adopted by Mendrek et al. for RARC [28–30]. Such new metrics will enable better comparison between centres, patient counselling and enable greater granularity for such complex surgery as in our current study. Interestingly, the readmission rate was relatively low at 7.5%, compared to 8.2% reported by Liedberg et al. [31].

Sixth, in our study, 9% of patients had bladder recurrence and 6% had distant metastasis. These findings are highly consistent with open UTUC series. Hemal et al. found no local recurrence in their series of 48 patients [10]. Yong et al. conducted a multicenter international analysis of 1718 patients and found that bladder cuff excision improves recurrence-free survival in the bladder. However, the study was inconclusive about the benefit of bladder cuff excision on oncological results [32]. In our study, we observed one local recurrence at the kidney site. However, in the study conducted by Hemal et al., almost 10% of patients in both arms experienced bladder recurrence and distant metastases. The authors reported the 5-year recurrence-free survival, cancer-free and overall survival in their laparoscopic LNU arm to be 90,4%, 95,2% and 85,7% respectively [10]. Campi et al. reported 20% ipsilateral upper tract recurrence after RASU, and 7.5% distant metastases after RANU [22]. We reported our data with mean follow-up of 26 months. While the Recurrence-free survival at a median follow up of 26 months in our study was 84,4%, our patients had similar cancer-specific 95,4% and superior overall survival at 94%. De Groote et al. found 4-year Overall survival (OS) of 66% and recurrence-free survival (RFS) and 53% at a median follow-up of 15 months [16].

Finally, our study mortality rate is 3% (2/66) patients. Our rate is higher than what has been reported by others [23]. However, it is of note that our data relies on a real-life cohort and that the large confidence interval is not suited for comparability. Moreover, this could be attributed to the extensive comorbidities in some of our patients. Another reason could be that a significant portion of our patients underwent surgery in almost palliative symptomatic settings due to persistent uncontrolled macrohematuria. Seisen et al. proposed, in their systematic review dealing with the safety of kidney-sparing surgery (KSS) for UTUC and comparing it to RANU, similar survival after Kidney sparing surgery (KSS) versus RANU only for low-grade and noninvasive UTUC when using endourological interventions [15]. Moreover, Ditonno et al. found comparable oncological results between RASU and RANU patients, with better preservation of renal function in patients whose kidneys were spared [33]. In our study, only 5 out of 55 patients (10%) were suitable candidates for RASU. Among these patients, two had high-stage tumours, T2 and T3. This finding is consistent with Seisen and colleagues’ suggestion that selected patients with high-grade and invasive upper tract urothelial carcinoma (UTUC) could benefit safely from this type of surgery when feasible.

Our study has limitations. Our analyses were performed retrospectively. To ensure the study reflected real-world scenarios, all consecutive patients from aforementioned three treatment groups were included, representing different UTUC procedures. Nonetheless despite that sample size, in comparison, 66 cases in 4 years in one center is indiciates a higher number for a single center compared to 78 cases over 10 years in 3 high volume robotic surgery centers [16]. In consequence future series are necessary to confirm our findings. Specifically, our findings still serve as a proof of feasibility and proof of favorable patient outcomes. Thus, we anticipate further widespread adoption of the techniques reported in our study. Additionally, it’s worth noting that the study was conducted in a high-volume robotic tertiary center. Therefore the findings may not apply to other centers with different surgical focus or different caseloads.

## Conclusion

Robotic surgery is a viable treatment option for urothelial carcinoma of the upper urinary tract. Our study didn’t come up with new information. Still, we hoped to enrich the existing scientific body of literature regarding the adoption of robotic surgery in treating UTUC across the globe. Prospective studies are warranted to confirm its proposed benefits.

## Data Availability

The datasets generated and/or analysed during the current study are not publicly available due to national regulations on personal data protection but are available from the corresponding author on reasonable request.

## Abbreviations

ONU: Open radical nephroureterectomy
CDC: Clavien-dindo complication
UTUC: Upper urinary tract carcinoma
RANU: Robot-assisted radical nephroureterectomy
RARC: Robot-assisted radical cystectomy
RASU: Robot-assisted segmental ureterectomy
uc: Urothelial carcinoma
LNU: Laparoscopic radical nephroureterectomy
LAD: Lymphadenectomy
LN: Lymph nodes
AC: Anticoagulation
ASA: American association of anesthesiology score
BMI: Body mass index
Hgb: Hemoglobin
PSM: Positive surgical margins
ccs: Cancer specific survival
RFS: Recurrence free survival
OS: Overall survival
br: Bladder recurrence
